# Global burden of head and neck cancer: Epidemiological transitions, inequities, and projections to 2050

**DOI:** 10.3389/fonc.2025.1665019

**Published:** 2025-09-25

**Authors:** Hao Sun, Mulin Yu, Ziyue An, Fangcheng Liang, Bowen Sun, Yuanlong Liu, Shenquan Zhang

**Affiliations:** ^1^ Department of Radiation Oncology, The First Affiliated Hospital of Zhengzhou University, Zhengzhou, China; ^2^ Department of Respiratory Medicine, The First Affiliated Hospital of Zhengzhou University, Zhengzhou, China

**Keywords:** head and neck cancer, global burden, disparities, epidemiology, GLOBOCAN database

## Abstract

**Background:**

Head and neck cancer (HNC) is the seventh most frequently occurring cancer worldwide. The incidence and mortality of HNC have been demonstrated to significantly impact human health and increase the global health burden. We assessed temporal trends via Estimated Annual Percentage Change (EAPC) across 36 countries (2008–2017). No study has yet focused on the latest epidemiological changes of GLOBOCAN 2022 in HNC and made predictions about the trends to 2050.

**Objectives:**

Integrate historical HNC records and project global incidence and mortality trends to 2050 to support region-specific prevention strategies.

**Methods:**

Data on HNC cases and deaths including rates for subtypes were gathered from GLOBOCAN 2022 (https://gco.iarc.fr). The age-standardized incidence and mortality rates per 100,000 person-years were calculated by the direct age standardization method based on the Segi-Doll World standard population. We counted EAPC in 36 countries over the last decade (2008-2017). Eight of these countries with different levels of development were also selected to observe in detail their annual trends from 1980 to 2017. Relationship between the Human Development Index (HDI) and the HNC subtypes was examined using linear regression.

**Conclusion:**

From 2008 to 2017, both the number of male HNC deaths and cases decreased. However, in 2022, the men’s and older people’s HNC burden is still heavier. According to the projection, future reductions in overall HNC incidence and mortality are expected to materialize only in the event of a decline in cases and deaths by 2 or 3 percent or more.

## 1 Introduction

Head and neck cancer (HNC) encompasses a broad spectrum of heterogeneous diseases, including lip, oral cavity cancer (ICD10 C00), oropharynx cancer (ICD10 C10), nasopharynx cancer (ICD10 C11), hypopharynx cancer (ICD10 C13), pharynx cancer (ICD10 C14), and larynx cancer (ICD10 C32). Risk factors for HNC vary based on tumor location. These include tobacco smoke exposure, smokeless tobacco, alcohol use, human papillomavirus (HPV), and Epstein–Barr virus. The epidemiological trend of HNC has increased significantly due to the increasing incidence of HPV -associated oropharynx cancer (1, 2). We assume that HNC is classified as a neoplasm with a high mortality rate mainly due to the paucity of available oncological treatments and the disease itself progression (1, 3). World Health Organization, in collaboration with different countries, has taken measures related to the typical risk factor for HNC. However, the effectiveness of the prevention and treatment measures for HNC implemented over the past decade, the latest 2022 data, and the potential reduction in the future burden of HNC have not yet been addressed in any paper.

We extracted estimated annual percentage change (EAPC) from the GLOBOCAN database for 36 countries over the period 2007-2018, and found significant changes. In 2022, HNC accounted for 480,000 fatalities and 940,000 new cases, making it the seventh most prevalent type of cancer globally, according to GLOBOCAN 2022 (4, 5). In developed countries, HPV related HNC has surpassed alcohol related disease. Significant differences have been identified in all aspects of HNC cancer prevalence and mortality due to environmental exposures, as well as differences in healthcare systems. Based on the speculation that a strong correlation between the prevalence of HNC and various socio-economic factors, including educational attainment and income levels. We conducted a stratified analysis of HNC burden by HDI levels. Analysis of the 2022 data reveals the latest susceptible populations for HNC and identifies the reasons why, so that more effective health policies can be developed. In terms of predictive analysis, we believe that the future outlook for HNC is not optimistic, and we have analyzed and predicted the situation up to 2050.

## 2 Materials and methods

### 2.1 Data sources

Incidence and mortality data for HNC were obtained from the GLOBOCAN 2022 database, a comprehensive global source for cancer burden estimates.

### 2.2 Statistical analysis

#### 2.2.1 Temporal trend analysis: 2007-2018

A total of 36 countries’ EAPC data were collected between 2007 and 2018. The EAPC describes the change in trend. It is calculated by fitting a regression line to the natural logarithm of the ASR, using the calendar year as a regressor variable. We then analyzed changes in HNC incidence and mortality rates in 36 countries, using 0% as a cut-off. Due to limited data availability prior to 1980s, a focused analysis was conducted on eight countries (four developed, four developing) with more complete historical records. Within these countries, the analysis specifically targeted individuals aged ≥50 years, a demographic previously identified as having higher HNC susceptibility (3). Sex-specific burden comparisons were performed across all included countries.

#### 2.2.2 Contemporary disease burden assessment: 2022

The study of this database examines numbers and rates in 2022 of 6 specific cancers classified by sex and age category covered 185 countries or districts (6). The age-standardized incidence rate (ASIR) and age-standardized mortality rate (ASMR) per 100,000 person-years were calculated from the 1966 Segi–Doll World standard population (7). Patients are stratified by sex and 5-year age groups (0–4, 5–9,…, 80–84, 85+ years). Regional disparities were analyzed according to the United Nations geographical regions and subregions.

We also measure burden using HDI outlined in the United Nations Development Program’s 2013 Human Development Report: Human Development Report 2013 | Human Development Reports (undp.org). HDI is a composite indicator of life expectancy, education, and gross domestic product. Based on the HDI groupings established by the United Nations in 2012, the 153 countries/areas included are categorized into four categories: very high (≥0.800), high (0.700-0.799), medium (0.550-0.699), and low (< 0.550) HDI. Linear regression was employed to quantify the association between HDI level and subtypes of HNC. Statistical significance was defined as p < 0.05. All required calculations were conducted in R (version 4.3.1).

#### 2.2.3 Projected burden through 2050

We provide projections of global HNC incidence and mortality in 2050, based on global population projections. Incorporate 7 scenarios into assumptions for annual mortality/incidence based on 2022 data: 1%, 2%, 3% increase; 1%, 2%, 3% decrease; or no change. Results will be presented graphically.

The assessment of future data is based on current data as of 2022. The key assumptions are that national rates, as estimated in 2022, do not change in the prediction period 2022–2050 and that the national population projections are correct for these years (8). The projections of current incidence rates, which can be found at https://gco.iarc.fr/today/en/data-sources-methods, incorporate a number of methodologies, primarily including: 55 countries use “national (or subnational with coverage exceeding 50%) projected incidence rates”; 22 countries use “latest incidence data from a single registry extrapolated to the 2022 population”; and 38 countries with missing data use “incidence data from neighboring countries or regional registries”. The methods used to predict the 2022 mortality rate mainly include: 90 countries using national rates projected to 2022, 21 countries using estimated national incidence rates by modelling, using mortality: incidence ratios derived from survival estimation, and so on. In terms of future population predictions, the national population estimates were derived from the latest United Nations population database (9). These data and methods can be accessed at https://population.un.org/wpp/.

## 3 Results

In the 2007–2018 data set, a decline in male prevalence and an increase in female prevalence were observed among the 36 countries. Crossing countries and regions., disparities in HNC mortality and incidence were noticed. In 2022, the highest rates were observed in South Central Asia and the lowest occurred in Central America regions; the incidence rate in Ireland and the mortality rate from Romania are the highest data separately in the 185 countries. Men have three to four times higher ASIR and ASMR rates (15.3 and 7.8 per 100,000 people, respectively) than women (4.6 and 2.1 per 100,000 people, respectively). It is projected that if rates remain stable as they were in 2022, there will be 1,600,000 new cases and 820,000 deaths from HNC in 2050.

### 3.1 Incidence and mortality over time

**Figure 1:**
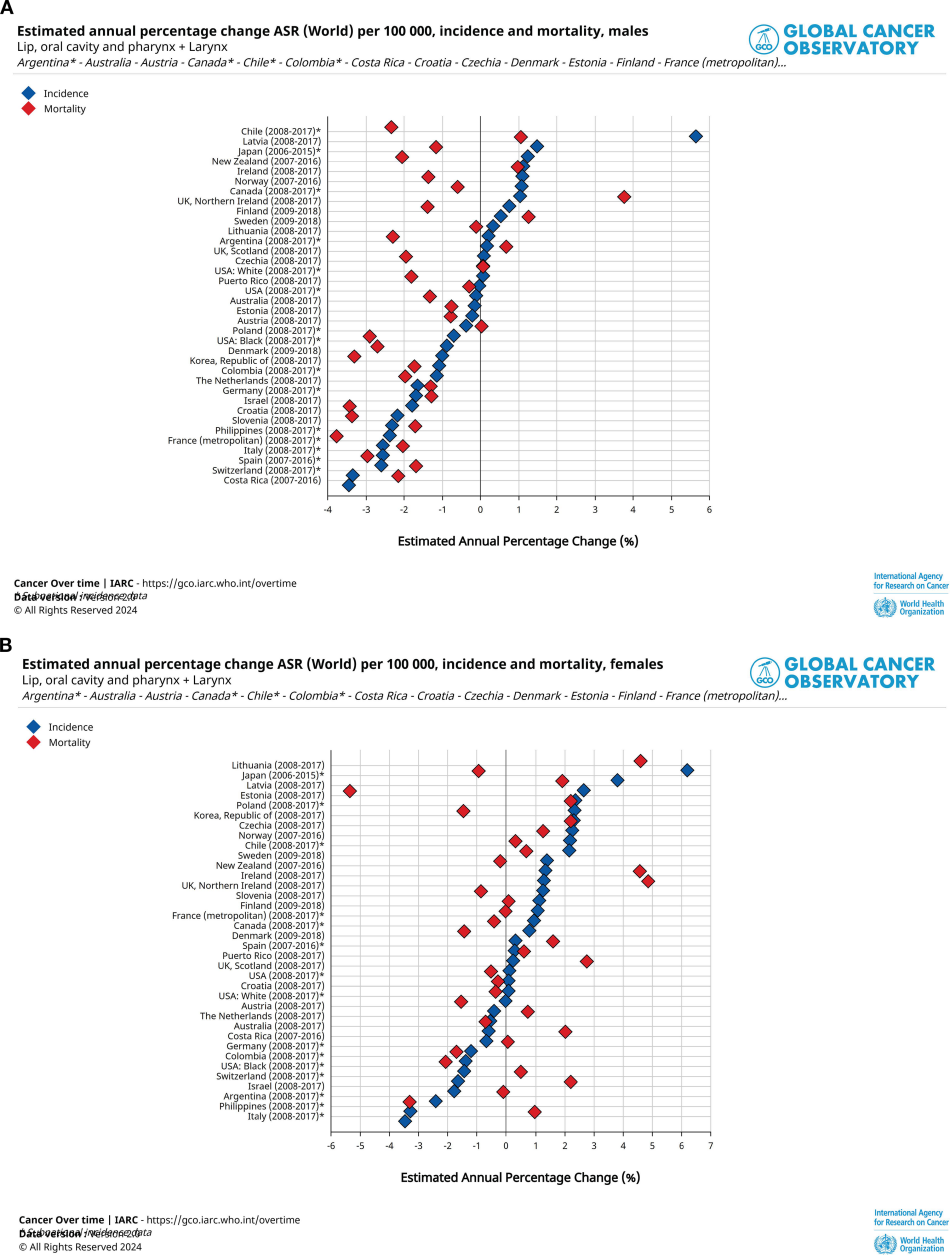
Estimated annual percentage change (EAPC) of head and neck cancer (HNC) across 36countries, 2007-2018. **(A)** EAPC of age-standardised rates (ASR) of occurrence and fatality among males. A black vertical line indicates no change (0%). A diamond to the left of it indicates a downward trend in HNC data for that country over the last ten years, and a diamond to the right indicates an upward trend. **(B)** EAPC of ASR in incidence and mortality rates among females. These demonstrate the effectiveness of the various preventive and therapeutic measures we have taken to address HNC over the past decade.

Between 2008 and 2017, EAPC of incidence in men has decreased in most countries (21 out of 36), while the EAPC of women has increased in 12 countries. Male EAPC declined in 29 out of 36 countries, while female EAPC mortality declined in 17 out of 36 countries. The country with the largest increase in male incidence was Chile and the largest decrease was Costa Rica, while the country with the largest increase was Lithuania and the largest decrease was Italy. Regarding mortality, the largest increase in male mortality was recorded in the United Kingdom, Northern Ireland and Ireland, and the largest decrease in France (metropolitan). The countries with the largest increase in female mortality were also the United Kingdom, Northern Ireland and Ireland, and the country with the largest decrease was Estonia. Among the 36 countries, the Philippines and Latvia had the lowest incidence EAPC (men: -3.54%, women: -5.83%) and the highest mortality EAPC (men: 0.88%, women: 3.47%) for both men and women. Meanwhile, Japan is the country with the highest incidence of EAPC for men (1%). The same is true for the Czech Republic, but for women the data is -2.87%. The lowest mortality EAPC appears in Colombia for men (-3.20%) and in the Republic of Korea for women (-4.82%). **Figure 1** visualizes the data.

**Figure 2:**
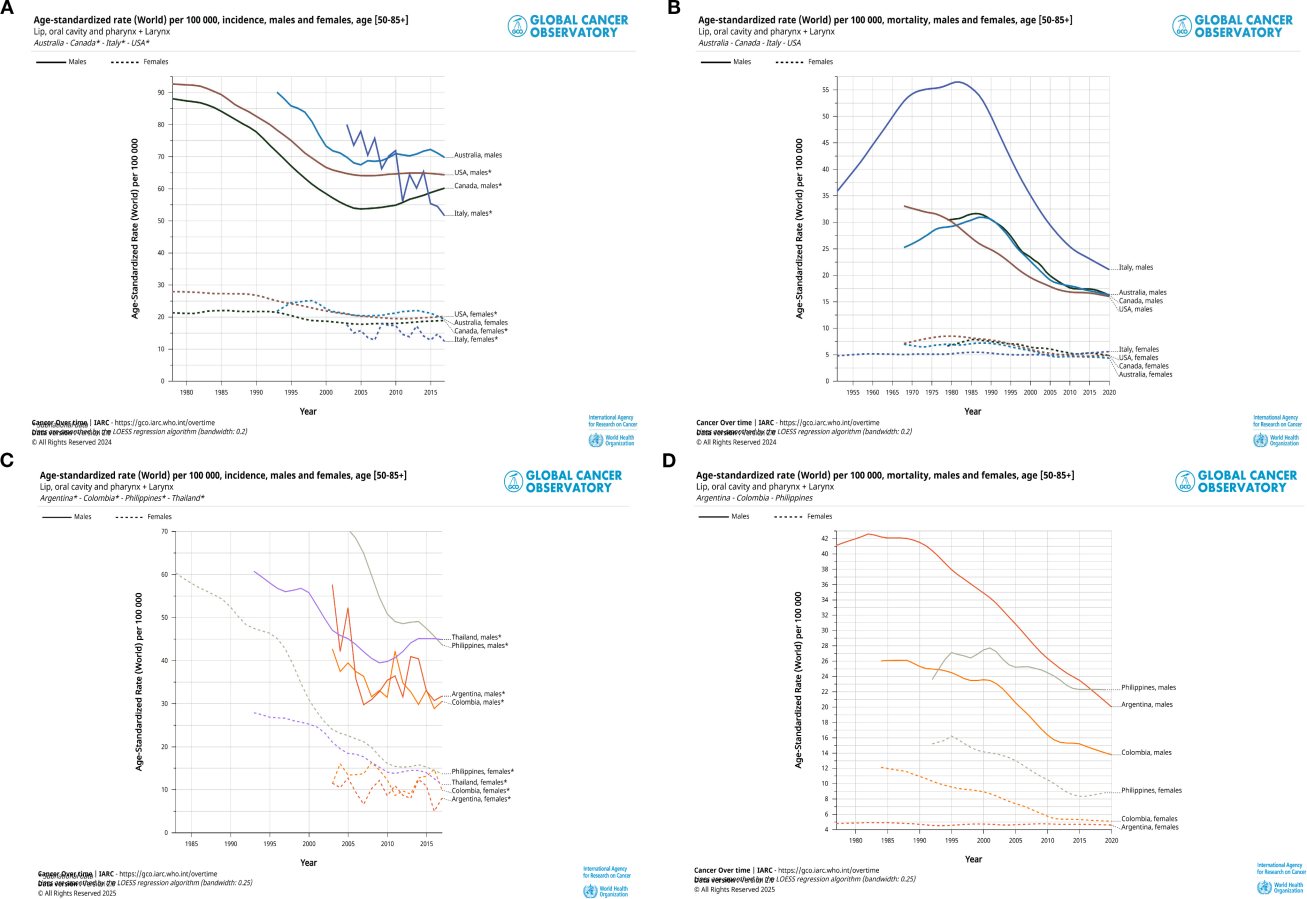
Trends in age-standardised rates (ASR) in selected developing or developed countries, 1985 to 2020. **(A)** Trends of incidence in ASR per 100–000 persons in the middle-aged and elderly (50–85 years) population in four developing countries (Thailand, Philippines, Argentina, Colombia), 1985-2020. **(B)** Trends of mortality in ASR per 100–000 persons in the middle-aged and elderly (50–85 years) population in three developing countries (Philippines, Argentina, Colombia), 1985-2020. **(C, D)** Trends in ASR per 100–000 persons in the middle-aged and elderly (50–85 years) population in four developing countries (Australia, Canada, ltaly and United States of America), 1985-2020. These graphs show a visualisation of the trends in the hrad and neck cancer (HNC) rates over the last 35 years between different countries and between the sexes.

Since the close of the 20th century, there has been a marked improvement in incidence and mortality trends for both genders. In the four developed countries, such as the United States, Australia, Canada and Italy, there has been a consistent decline in incidence rates since 1985, from approximately 90 per 100,000 people to around 65 per 100,000 people. A similar trend has been observed in four developing countries (Thailand, the Philippines, Argentina and Colombia), where rates have fallen from 65 per 100,000 people to 45 per 100,000 people. The prevalence has remained stable and low, and has declined from approximately 25 to 20 in all four developed countries, with the greatest fluctuation in Italy. The incidence has declined more sharply over decades in these four developing countries, paralleling the downward trend for males. In the period spanning from 1985 to 2020, a notable decline in mortality rates was observed among males in the four developed countries (Italy, Australia, Canada, and the United States). A similar decline was also evident in the three developing countries (the Philippines, Argentina, and Colombia). The analysis revealed a consistent downward trend in HNC mortality rates among both males and females, with the exception of Argentina (**Figure 2**).

### 3.2 Incidence and mortality for now

**Figure 3:**
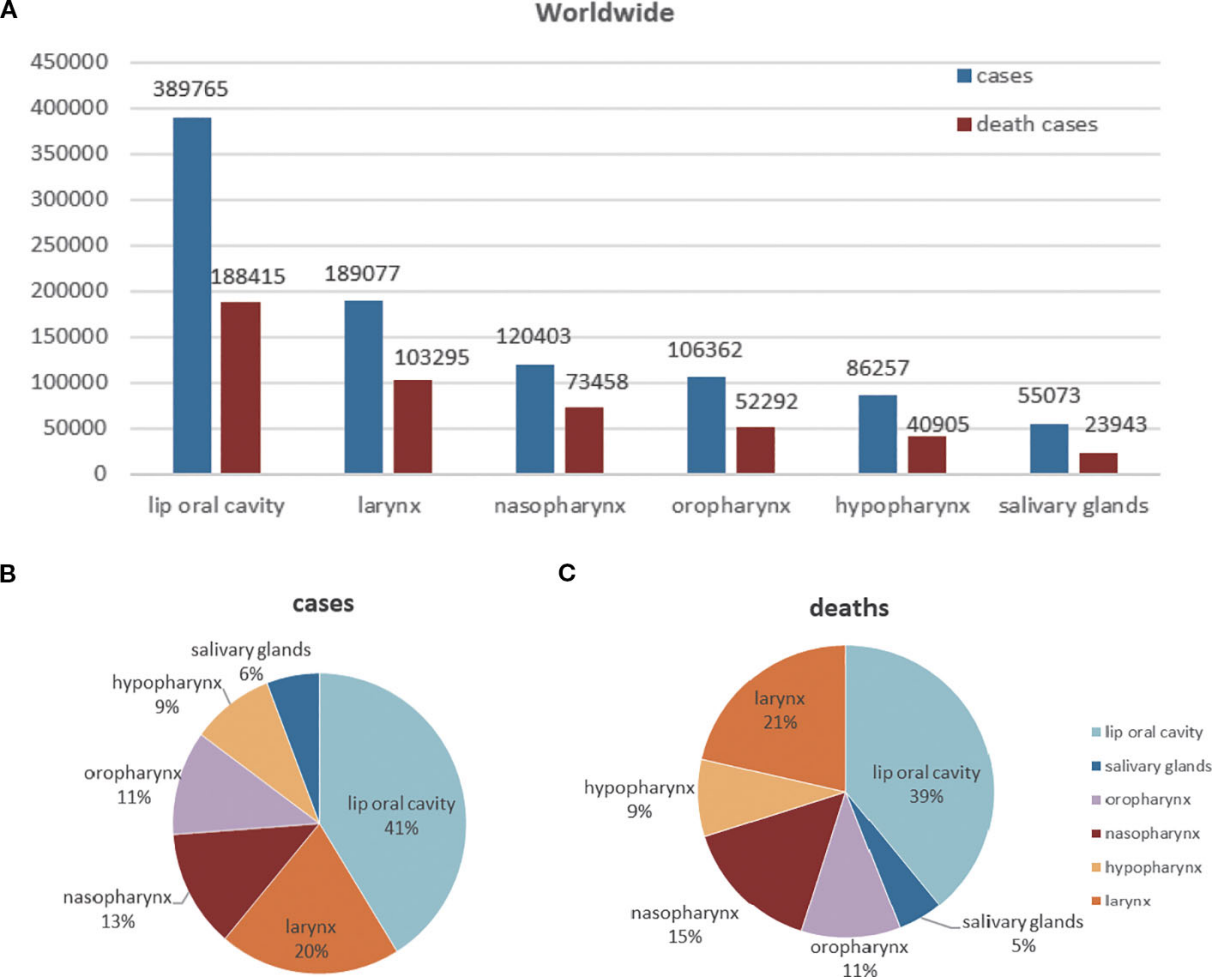
Cases and deaths of different head and neck cancer (HNC) and their proportions in 2022. **(A)** The total global burden of the respective type of HNC on the given geographic area in 2022. **(B, C)** Cases and deaths proportion by HNC subtype for all ages as a share of the entire number of HNC cases or deaths. These graphs visually illustrate the distribution of different HNC and clearly show the magnitude of the public health burden of each subtype.

In 2022, a total of 947,211 new cases of HNC were diagnosed, constituting 4.7% of all cancer cases. Moreover, 482,428 deaths were attributed to HNC, accounting for 4.9% of all cancer-related mortalities (**Figure 3A**). The age-standardized rate (ASR) of HNC incidence and mortality were 9.8 and 4.9 per 100,000 people worldwide, respectively. The most prevalent subtypes were lip, oral cavity cancer (41% [389,846 cases]) and larynx cancer (20% [189,077 cases]). Consistent with the case data, larynx cancer ranked second among HNC deaths, accounting for 21% of the total. Salivary gland cancer was the least prevalent neoplasm among HNC, comprising approximately 6% of these cancers (55,073 cases). The distribution of HNC burden by subtypes is depicted in **Figures 3B, C**.

#### 3.2.1 Geographical differences by UN regions

**Figure 4:**
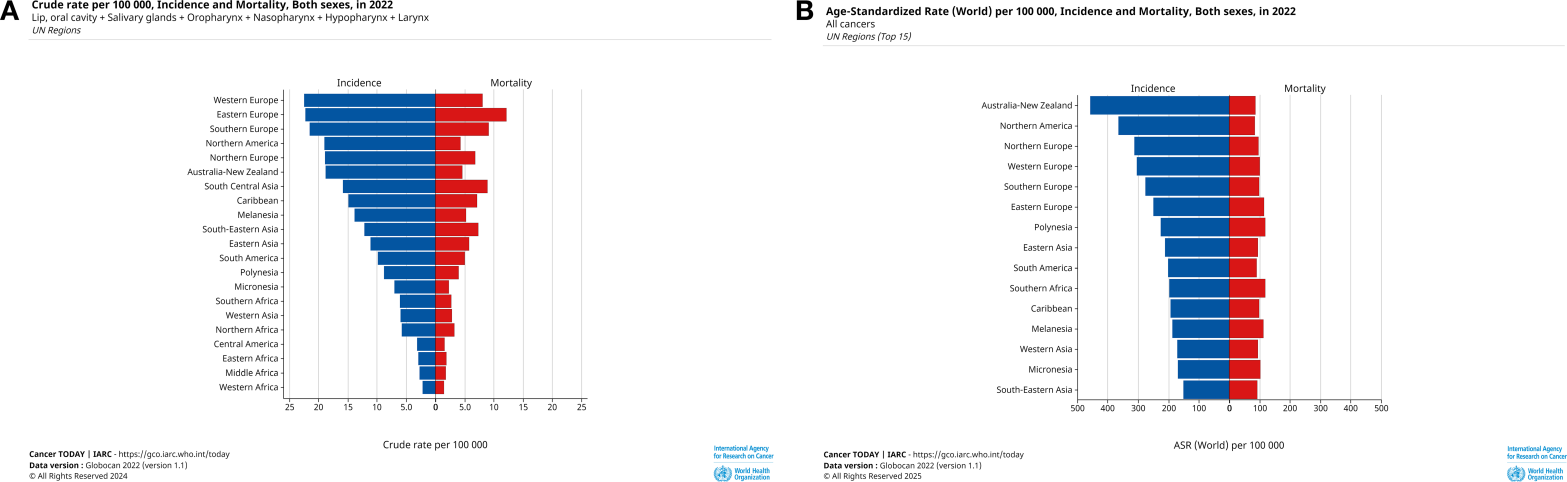
Cases and deaths from HNC in 2022 for all age groups and ASR by UN region. **(A)** Crude cases and deaths for HNC in 2022, numbers of cases are shown in descending order of crude cases, **(B)** ASR of HNC for all ages in 2022, incidence rates are shown in descending order of incidence rates. These graphs provide a visual breakdown of HNC by UN region, clearly illustrating the extent of HNC harm in different regions and providing geographic specificity for developing prevention and screening for HNC.

**Figure 5:**
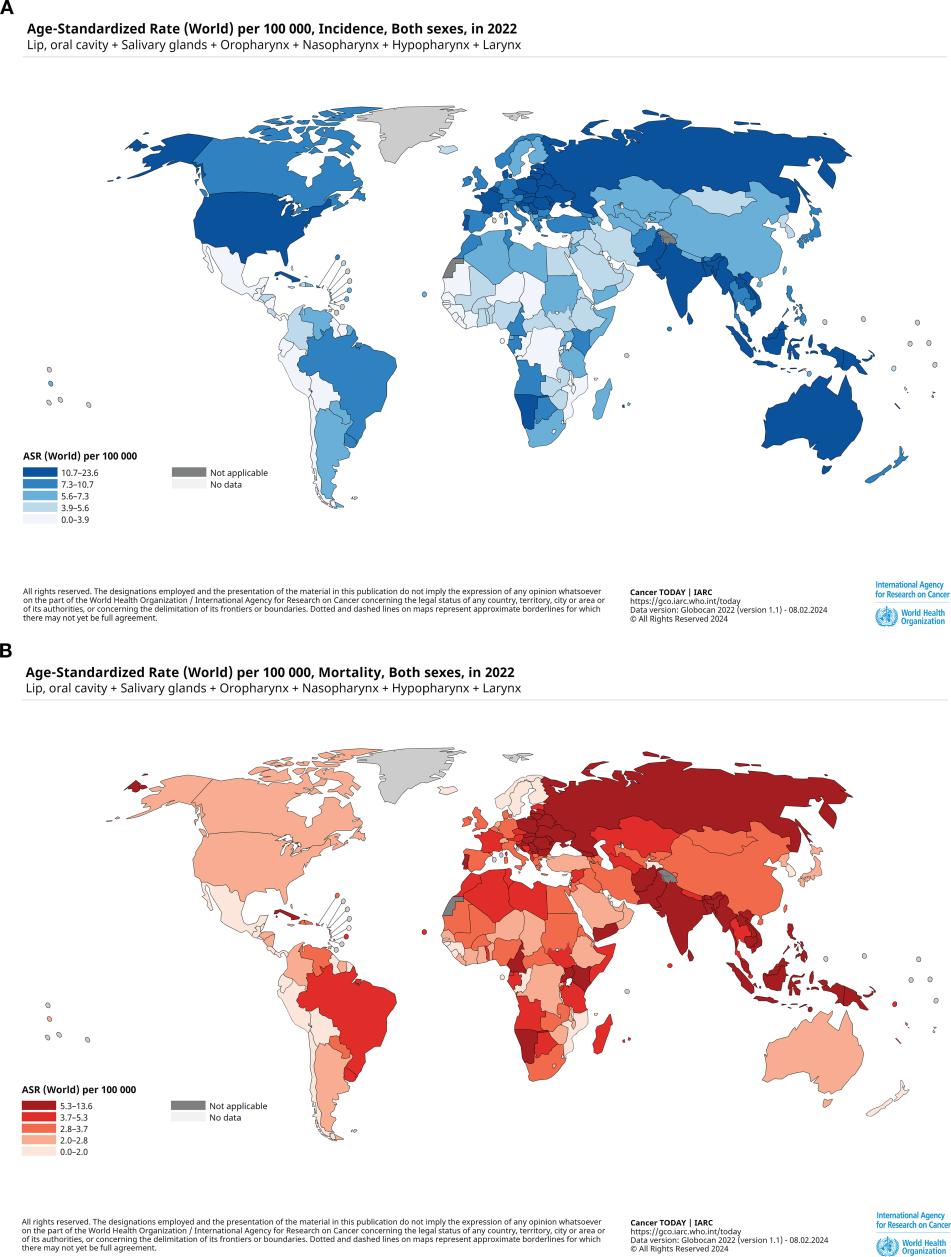
Worldwide age-standardised rates (ASR) per 100,000 people of head and neck cancer (HNC) both sexes for all ages in 2022. **(A)** Age-standardised incidence rates (ASIR) of HNC per 100,000 people of the population across all age groups in 2022. **(B)** Age-standardised mortality rates (ASMR) of global HNC per 100,000 people of the population across age groups. The global hotspots of infection and mortality are clearly highlighted by these images.

Melanesia had the highest incidence rate (ASR=18.5, cases=1596) and the second highest mortality rate (ASR=7.4, deaths=607). While in South Central Asia, we observed the highest mortality rate (ASR=9.2, deaths=183867) and incidence rate (ASR=16.2, cases=326700) of it is after Melanesia. Central America had the lowest incidence (ASR=2.9, cases=5741) and mortality (ASR=1.4, deaths=2936) (**Figure 4**). UN region incidence and mortality rates are shown in **Figure 4**, and geographical differences are shown in **Figure 5**.

**Figure 6:**
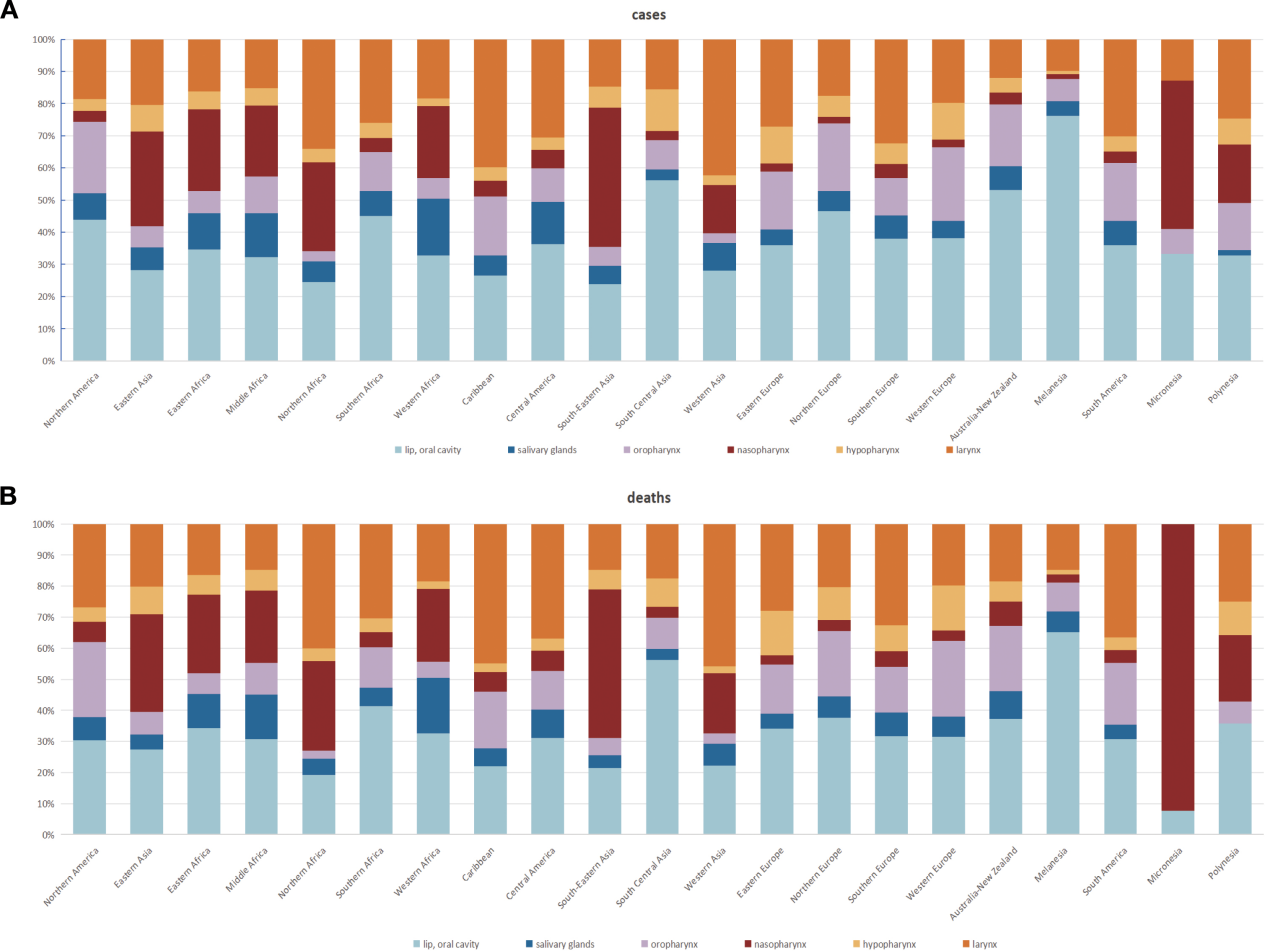
Proportion of different types of head and neck cancer (HNC) among new instances and fatalities **(A)** The proportion of lip, oral cavity, nasopharynx, oropharynx, hypopharynx and larynx among patients that are associated with HNC. **(B)** Proportion of cases in which HNC was the cause of death. These show how common and deadly each type of HNC is, and what type is contributing most significantly to the general burden.

In most regions, oropharyngeal cancers represent more than one-third of all cases, higher than other subtypes, such as in North America (44%), Southern Africa (45%), and Northern Europe (47%), while in some regions, such as Australia and New Zealand (53%), South Central Asia (56%), and Melanesia (76%), the proportion of oropharyngeal cancers is more than half. Regions and countries with high rates of death and occurrence for lip, oropharyngeal, and oral cancers often report relatively low incidence rates for hypopharyngeal cancers (**Figure 6A**). However, in Micronesia, Southeast Asia, and East Asia, the proportion of nasopharyngeal cancers is more than 30%, surpassing the proportion of the lip and oral cavity cancers. Among the six subtypes, Western Asia has the highest proportion of laryngeal cancer incidence (42%), and South-Central Asia has the highest proportion of hypopharyngeal cancer incidence (13%). In terms of mortality in different regions, deaths from lip, oral cavity, and nasopharynx cancers are often higher (**Figure 6B**).

#### 3.2.2 Geographical differences by countries

**Figure 7:**
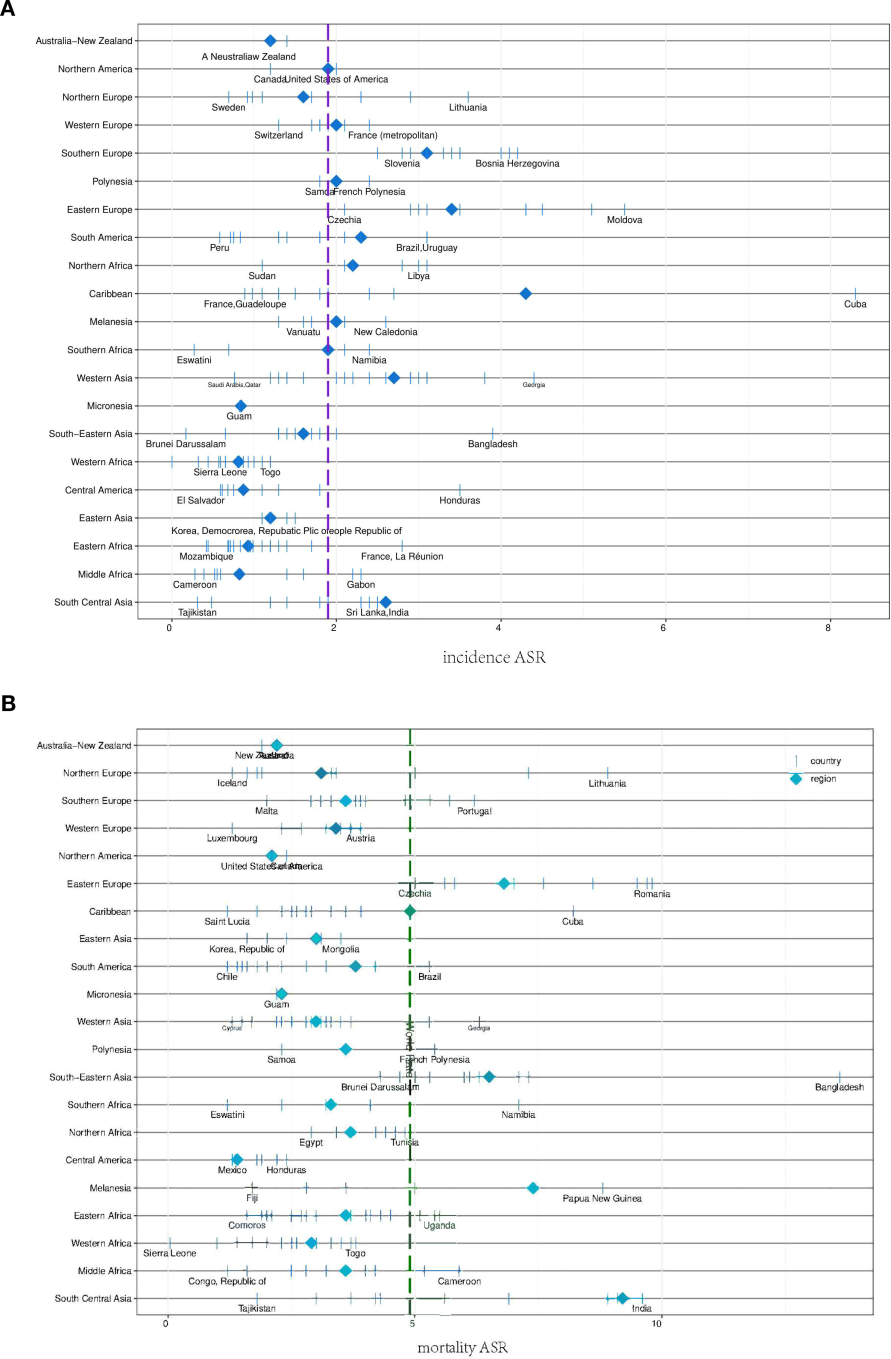
Age-standardised incidence rates (ASIR) and age-standardised mortality rates (ASMR) for head and neck cancer (HNC) among people in different regions of the world. **(A, B)** ASIR are displayed above and mortality rates for HNC among people by regions of the world are displayed below. The diamonds represent the incidence (blue-violet) and mortality (green) rates for these regions. The purple vertical line represents incidence and the green vertical line represents mortality. This graph highlights regional differences in the burden of HNC, highlighting areas with particularly high incidence and mortality.

The incidence rate in Ireland reached 9.7 per 100,000 people, and the mortality rate in Romania reached 9.8 per 100,000 people, which are both the highest data among the 185 countries. The highest number of HNC cases was found in five countries: India (247,924 cases), China (145,603 cases), the United States (63,440 cases), Bangladesh (36,808 cases), and Indonesia (33,603 cases). The highest number of HNC deaths were recorded in five countries: India reported the highest incidence rate for lip, oral cavity cancer at 9.9 per 100,000 people, with 143,759 cases. For cancer of the salivary glands, Solomon Islands has the greatest mortality rate at 1.2 per 100,000 (deaths = 7), while Togo has the highest incidence rate at 1.5 per 100,000 (cases = 77). Slovakia has the highest incidence rate of oropharyngeal cancer, at 5.1 per 100,000 (cases=458). The greatest incidence and fatality rate of nasopharyngeal cancer (7.5 and 5.0 per 100,000 individuals) are observed in the Maldives. In comparison, highest rates of oropharyngeal cancer incidence (5.5 per 100,000 people, cases= 8491) and incidence (2.4 per 100,000 people, deaths=3555) happen in Bangladesh. With 8.3 per 100,000 people (cases=1846) and 4.0 per 100,000 people (deaths=952), Cuba ranks highest for laryngeal cancers occurring and dying. As illustrated in **Figure 7**, the incidence of HNC is typically higher in nations in other areas than in other nations. These figures reveal significant geographical differences in the incidence and fatality rates of HNC globally. HNC incidence is notably high in countries in Eastern Europe, Southeast Asia, and Melanesia, and most countries in these regions have mortality rates above the global average.

#### 3.2.3 Differences between male and female

In 2022, the global incidence of HNC reached 947,211 cases, with 130,808 cases affecting men and 20,847 cases affecting women. The male-to-female ratio of the ASR of HNC is 2 to 3 times higher. The ASIR and ASMR of larynx cancer in males (ASIR=1.88 per 100,000 people, ASMR=3.51 per 100,000 people) is 7–8 times higher than that in females (ASIR=0.23 per 100,000 people, ASMR=0.33 per 100,000 people). **Table 1** shows HNC incidence and mortality statistics for males (chart a) and females (chart b), age-standardized to the world’s standard population.

**Table 1:** Male (chart a) and female (chart b) head and neck cancer (HNC) -specific incidence and mortality data.

a						
Rates	Label	ICD code	Number	ASR (world)	Crude rate	Cumulative risk
incidence	Lip, oral cavity	C00-06	268999	5.8	6.8	0.67
	Salivary glands	C07-08	30963	0.66	0.78	0.07
	Oropharynx	C09-10	86339	1.9	2.2	0.23
	Nasopharynx	C11	86289	1.9	2.2	0.21
	Hypopharynx	C12-13	72077	1.6	1.8	0.19
	Larynx	C32	165794	3.5	4.2	0.44
mortality	Lip, oral cavity	C00-06	130808	2.8	3.3	0.32
	Salivary glands	C07-08	13989	0.29	0.35	0.03
	Oropharynx	C09-10	42818	0.91	1.1	0.11
	Nasopharynx	C11	54104	1.2	1.4	0.14
	Hypopharynx	C12-13	34564	0.73	0.87	0.09
	Larynx	C32	90384	1.9	2.3	0.23
b						
incidence	Lip, oral cavity	C00-06	120847	2.3	3.1	0.26
	Salivary glands	C07-08	24120	0.49	0.62	0.05
	Oropharynx	C09-10	20061	0.39	0.51	0.05
	Nasopharynx	C11	34145	0.73	0.87	0.08
	Hypopharynx	C12-13	14180	0.29	0.36	0.03
	Larynx	C32	23397	0.45	0.60	0.05
mortality	Lip, oral cavity	C00-06	57630	1.1	1.5	0.12
	Salivary glands	C07-08	9953	0.18	0.25	0.02
	Oropharynx	C09-10	9487	0.18	0.24	0.02
	Nasopharynx	C11	19378	0.39	0.50	0.04
	Hypopharynx	C12-13	6338	0.12	0.16	0.01
	Larynx	C32	12975	0.23	0.33	0.03

Chart a. HNC-attributable incidence and mortality date in male, including number, age-standardised rates (ASR), crude rate and cumulative risk. Chart b. HNC-attributable incidence and mortality date in female, including number, ASR, crude rate and cumulative risk.

#### 3.2.4 Differences between different age groups

**Figure 8:**
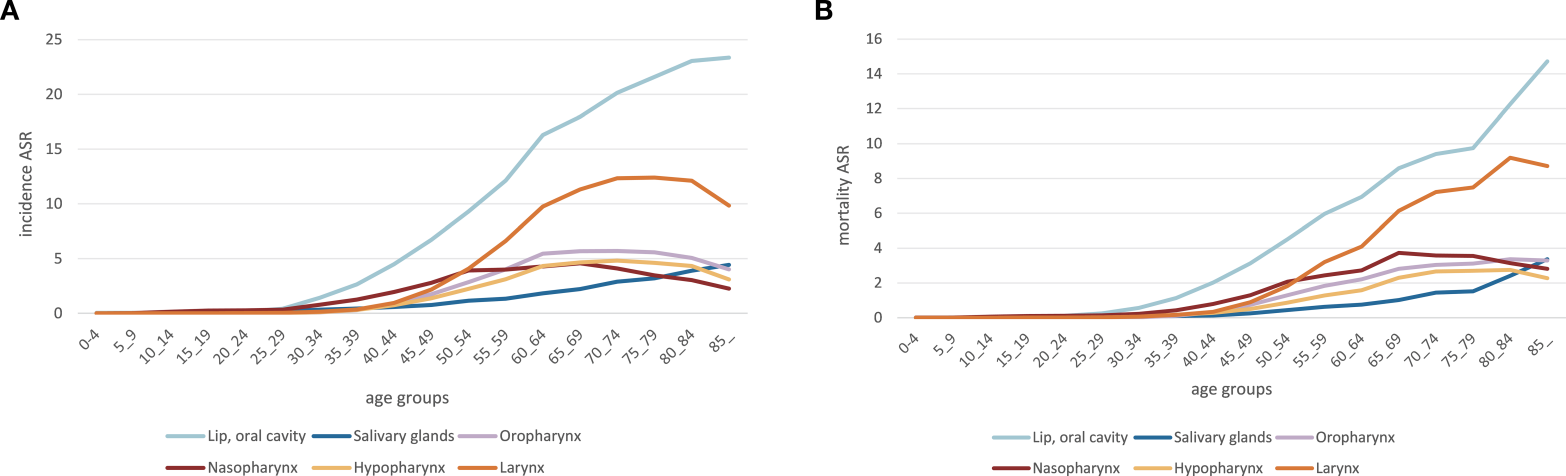
Incidence and mortality rates of head and neck cancer (HNC) according to age-standardised rates (ASR) in age groups. **(A)** HNC prevalence expressed as ASR. The distribution of new HNC cases by age group is displayed in this graph, which sheds light on the age groups most impacted. **(B)** HNC mortality rates for the same age categories, also displayed as ASR. The distribution of HNC-related mortality by age group is displayed in this graph, highlighting the most vulnerable groups.

From infancy to the elderly, a wide range of age groups are affected by HNC, with the highest incidence observed in individuals between 50–54 years old (138,813 cases) and the most significant mortality rate seen in those between 60–64 years old (131,666 deaths), primarily due to lip and oral cavity tumors. The disease manifests with minimal visibility in individuals younger than 30, and its prevalence increases with age. Concurrently, no apparent mortalities occur before the age of 35. The highest incidence (ASR=50.9) of HNC is documented in the 75–79 age group, while the highest mortality (ASR=35.2) is observed in the over 85 age group. For larynx cancer, the incidence exhibits a slight decline after the age of 75. Conversely, the mortality rate for the six subtypes of HNC exhibits an upward trend with age, with no discernible decrease. The relationship between age and incidence, as well as mortality, is illustrated in **Figure 8**.

#### 3.2.5 Association of incidence and mortality with HDI

**Figure 9:**
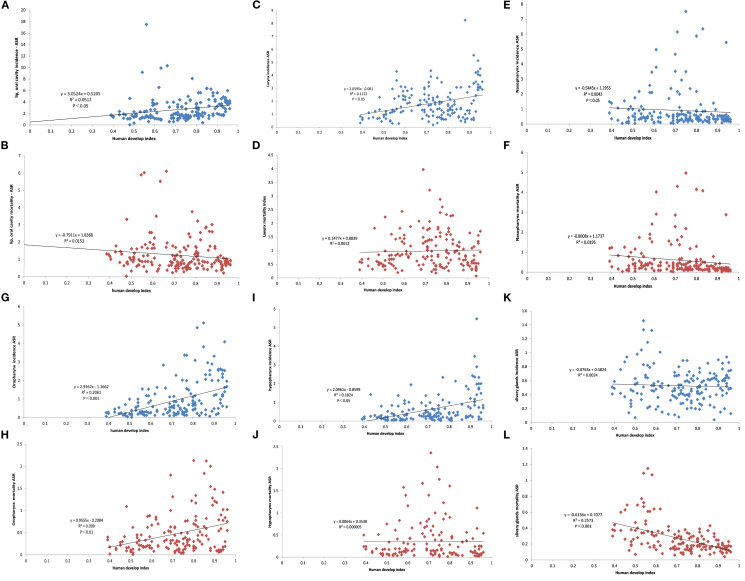
Findings on the link between head and neck cancer (HNC) subtypes and the Human Development Index (HDI). **(A, C, E, G, I, K)** Scatterplot showing the linear correlation between the incidence rates of each type of HNC and HDI in different countries. The regression line (solid black line) represents the best-fit linear relationship. **(B, D, F, H, J, L)** Scatterplot showing the linear correlation between each type of HNC mortality rates and HDI. This analysis provides insight into how socio-economic factors, as reflected in the HDI, influence both the incidence and mortality of HNC.

Of the total number of cases, 326,175 were documented in countries with moderate HDI, 293,426 in those with very high HDI, 277,810 in countries with high HDI, and 49,526 in those with low HDI. With regard to the incidence rate of HNC, moderate HDI countries exhibited the highest rates (15.1 per 100,000 people), followed by very high HDI countries (9.9 per 100,000 people). The lowest rate was found in countries with a low HDI (6.9 per 100,000 people). A similar trend was observed in the mortality rate of HNC, with moderate HDI countries (8.6 per 100,000 people) again exhibiting the highest rates, followed by low HDI countries (4.7 per100,000 people). Countries with a very high HDI exhibited a notably low mortality of 3.5 per 100,000 people. As illustrated in **Figures 9**, the relationship between the rates for different HNCs and HDI is demonstrated. Analysis of variance showed a slightly negative correlation between salivary gland cancer mortality and HDI, while oropharynx cancer incidence and mortality exhibited a positive association with HDI. Additionally, lip, oral larynx, and hypopharynx cancer incidence demonstrated a positive link with HDI. Negative relationships were identified between the incidence of nasopharynx cancer and HDI.

### 3.3 Incidence and mortality projections to 2050

**Figure 10:**
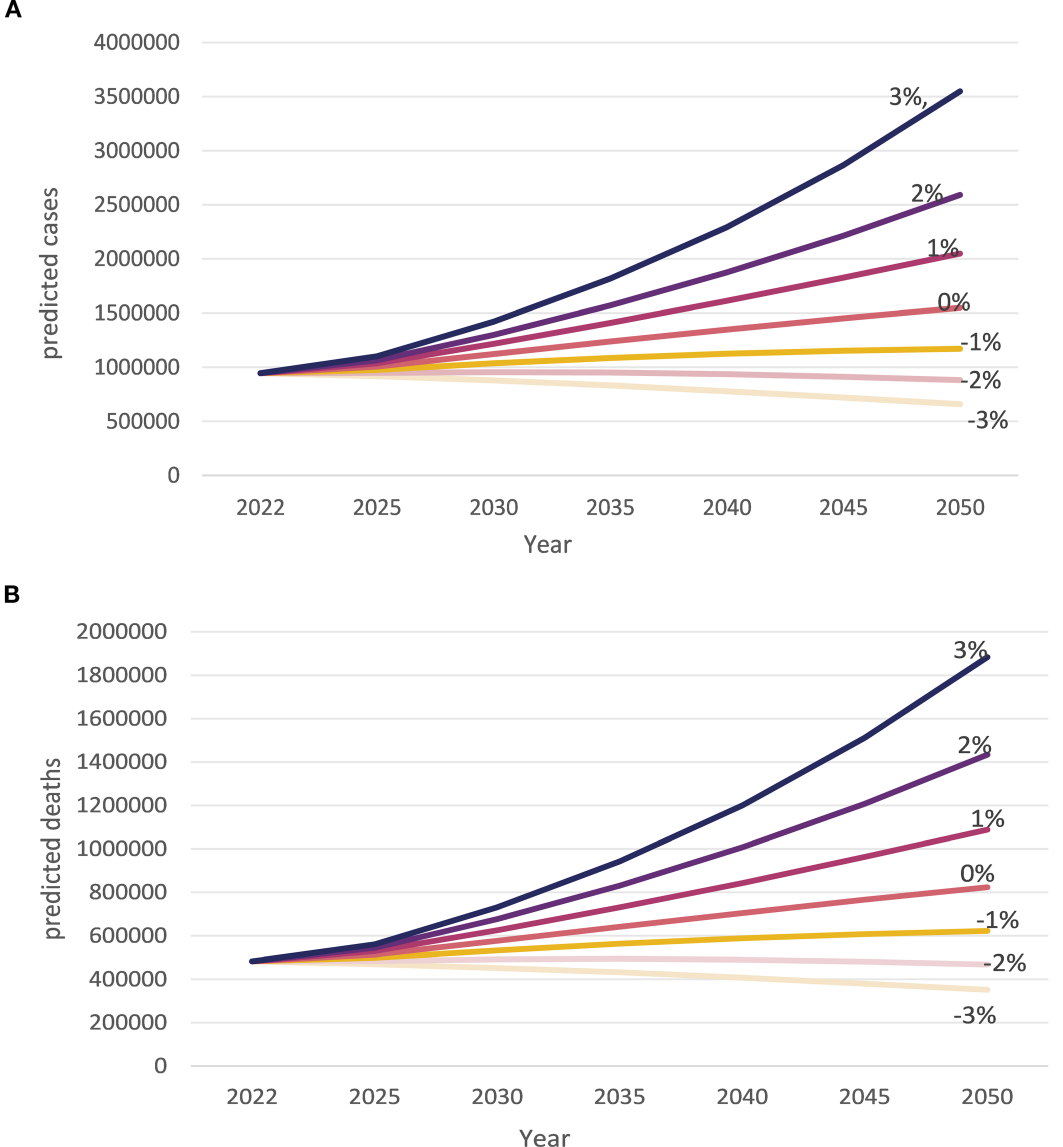
Projected new cases and deaths from head and neck cancer (HNC) in 2050. **(A)** The projected number of new cases of HNC in 2050. The graph provides a detailed trend. It highlights the expected distribution of new cases in the future. **(B)** The projected number of deaths from HNC in 2050. The graph provides a comprehensive overview of the future trend in deaths. They highlight the estimated death toll and the proportionate share of HNC in the total mortality burden.

Should the current trend continue, it is projected that the number of illnesses and associated deaths will climb to 1,550,796 new cases and 823,471 deaths in 2050. representing an increase of 1.6 and 1.7 times the 2022 figures, respectively. Furthermore, should these trends persist, the number of new diagnoses is projected to surpass one million in 2025. Projections indicate that new cases and deaths could reach 3,548,110 (3.7 times the 2022 figures) and 1,884,042 (3.9 times the 2022 figures), respectively, by 2050, under a scenario of a three percent annual increase. Projections indicate that the number of new cases and deaths of HNC will increase by 2050 under scenarios where the annual rate increases by less than -1%, 0%, 1%, 2%, or 3%. As demonstrated in **Figure 10**, both panels a and b illustrate that the global epidemiological burden of HNC will be mitigated only when the prevalence and mortality of HNC are diminished by 2% and 3%, respectively.

## 4 Discussion

### 4.1 Past trends (2007-2018)

The number of HNC cases has increased, but we have seen a decrease in overall incidence and mortality rates from 2007 to 2018. There’s been an uptick in female cases and a decline in male, narrowing the gender gap. This is consistent with the previous geographic study of Spain’s (10), and the results remain consistent when the scope is expanded to 36 countries. The ASR of HNC decreased after 1995 not only in some European countries, but also in countries usually with a high HNC prevalence, such as Philippines. This trend is more pronounced in the male population. This variation may be due to increased exposure of women to risk factors associated with cancer development (11). We believe that tobacco consumption, the highest risk factor for HNC (12), reveals the cause of this phenomenon. In accordance with economic progress, there has been an increase in tobacco use in developing countries. Conversely, there has been an overall decline in the prevalence of tobacco use in developed countries (13). Research has demonstrated that smoking increases the risk of HNC by approximately five times in men and twelve times in women. Furthermore, heavy alcohol consumption (three or more drinks a day) doubles the risk (14, 15).

The gender and geographic disparities extend beyond mere behavioral choices and are influenced by a complex interplay of socioeconomic and cultural factors. In many regions (for example, Portugal), targeted tobacco marketing directed towards women, in conjunction with increasing economic independence and evolving social norms, has contributed to their increased exposure (16). Conversely, successful public health campaigns in developed nations have predominantly reached male populations first, leading to a decline in their rates. Comprehension of the underlying causes of this issue is essential for the formulation of effective public health interventions (17, 18). Squamous cell carcinoma of HNC is not a legally guaranteed managed malignancy in Chile. The public health system and treatment availability and timeliness may have increased the burden of HNC in Chile (19). In contrast, Costa Rica exemplifies how robust public health policies reduce HNC burden. We attribute Costa Rica’s significant decline in HNC incidence to the rigorous implementation of risk-factor-specific interventions. The government initiated tobacco control efforts as early as the 1980s (20), culminating in the enactment of the strict Law No. 9028 for Tobacco Control and its Harmful Effects on Health in 2012 (21). This law, one of the strictest anti-tobacco regulations in the world, established 100% smoke-free environments, imposed a total ban on tobacco advertising, and mandated prominent health warnings on packaging (22). In addition, actions have been taken to reduce alcohol consumption (23), and we believe that it is these public health measures, which are closely linked to HNC risk factors, that have made this country one of the most significant declines in HNC mortality among the 36 countries.

The comparison of these two cases demonstrates the impact of public health measures implemented by the government on the burden of HNC. While the incorporation of HNC within the framework of national cancer control strategies, coupled with the implementation of comprehensive legislative measures pertaining to tobacco and alcohol, is of paramount importance. It is recommended that policymakers give priority to the ratification and implementation of the WHO Framework Convention on Tobacco Control. In addition, N resources should be allocated to public awareness campaigns focusing on HNC risk factors and early symptoms, with interventions tailored to high-risk populations. Furthermore, investment in the enhancement of healthcare infrastructure is imperative to ensure the timely diagnosis and treatment of patients, thereby mitigating the adverse effects of delays within the health system. A promising downward trajectory in mortality rates of HNC has been observed across nations. This decline can be attributed, in large part, to ongoing exploration of tumor treatment and prevention strategies, complemented by the advancement of medical care on a global scale. The field has witnessed significant advancements in radiotherapy, as well as the combined utilization of radiosensitizing systemic therapy and chemoradiotherapy, resulting in improved survival rates for patients diagnosed with HNC, particularly those afflicted with HPV-associated oropharyngeal carcinomas (24).

In the global context of HNC, squamous cell carcinoma (SCC) of the lip and oral cavity constitutes a significant health concern (25). This condition manifests predominantly in developing countries, a prime example being Brazil (26). Conversely, the United States has witnessed a decline in the incidence of SCC of the oral cavity over the past decade (27). Regarding earlier investigations, a significant decrease in oral cancer trends has been found in high-income nations throughout the past three decades, driven by a shift in the distribution and prevalence of major risk factors (28, 29). This shift can be attributed to alterations in major risk factors (30).

### 4.2 Current status (2022)

The corresponding incidence and mortality rates vary widely in 2022 between geographical areas, reflecting the potential prevalence and distribution of key risk factors and specific differences in early detection and treatment in different countries and regions. Large differences between the sexes also emerged in 2022. The burden of HNC was three to four times higher in men than in women.

It is important to note the rising correlation between HNC and viral infections in certain developed countries, exemplified by a 225% increase in the incidence of HPV-positive oropharyngeal SCC in the United States between 1988 and 2004, a period during which the incidence of HPV-negative cancers declined by approximately 50% (5). HPV-associated HNC affects the larynx and hypopharynx more often than the oral cavity and is linked to a longer survival time among patients (13). Furthermore, there is a notable increase in HPV prevalence in the oropharynx, from 40.5% before 2000 to 72.2% in 2009 (31). Recent studies suggest a potential acceleration of the increase toward HPV-driven HNC in some developed nations (2, 32). A study in the United States revealed that, the annual number of cases of oropharyngeal cancer has already surpassed that of cervical cancer, and it is projected to exceed three times the number of cases by the year 2030 (24). And the International Agency for Research on Cancer and the US National Cancer Institute, convened the fourth Cancer Seminar meeting in November 2018 to focus on HPV-positive oropharynx cancer (33).

With real-world evidence already showing its impact in reducing the prevalence of oncogenic oral HPV infections, vaccination is pivotal to curtailing the rising incidence of HPV-positive oropharyngeal cancer (33, 34). However, global HPV vaccination efforts have been significantly hampered by the COVID-19 pandemic, which caused a documented decline in uptake of HPV vaccines during the pandemic (69.59%) and in the post-pandemic period (76%), as compared to the pre-pandemic (89.92%) era (35). This setback exacerbates existing health disparities, as low-HDI regions, which bears a high burden of HPV, face profound challenges in preventing and managing HPV-linked head and neck cancers due to limited resources (36).

This context of unequal global access makes the situation in high-HDI countries particularly critical for discussion. Although the countries with high HDI typically have well-established screening programs and high HPV vaccine coverage in target populations, they are not immune to challenges (37). Vaccine hesitancy, fueled by misinformation, and persistent socioeconomic and gender-based coverage gaps prevent these nations from achieving optimal herd immunity (38). Therefore, in these high-resource settings, there is an urgent need to integrate vaccination advocacy more effectively within screening programs and to launch public health campaigns specifically educating about the risk of HPV-driven HNC. Addressing these issues is vital to implementing a comprehensive prevention strategy that includes vaccination, screening, and novel treatments to reduce the overall burden of HPV-associated cancers.

Additionally, nasopharyngeal tumors are more likely to be infected with the Epstein-Barr virus, which is particularly common in Asia (13).

In addition to the well-established causative factors, recent research has identified a number of novel risk factors that contribute to the development of HNC. Among these factors, diet and nutrition have garnered significant attention, particularly in the context of non-smokers (39). A recent study conducted in 2023 established a correlation between high systolic blood pressure and the occurrence of HNC (12). This mounting evidence underscores the need for concerted efforts by public health organizations worldwide to devise novel strategies and precise objectives for the prevention and management of these malignancies.

It has been observed that the occurrence of lip and oral cavity. SCC shares sun exposure as a risk factor. However, tobacco and alcohol consumption, as well as viral infection, have also been identified as risk factors for mucosal lip and oral cavity cancers (26). Alcohol consumption has been demonstrated to be associated with an increased risk of developing oral cancer, particularly in conjunction with tobacco smoking (40). Furthermore, it is crucial to acknowledge the significant role of smokeless tobacco products as a major risk factor for oral cancer. According to previous studies, smokeless tobacco is most popular in Southeast Asia (41, 42), with South Central Asia accounting for 87.8% of the world’s total cases of oral cancer attributable to smokeless tobacco or areca nut (43). Evidence from a systematic review conducted in South-East Asia directly indicates a combined odds ratio of 4.7 for oral cancer among users of these products (44). The high prevalence of smokeless tobacco use is a key driver of the elevated burden of oral cavity cancers in South Central Asia. It contributed significantly to the region having the highest overall incidence and mortality rates of HNC globally in 2022.

The following risk factors have been associated with the incidence of laryngeal cancer: smoking, alcohol consumption, a poor diet, obesity, diabetes, hypertension, chemical workplace exposure, gastro-esophageal reflux, and dyslipidemia (45). Nasopharyngeal cancer has been found to be strongly associated with Epstein-Barr virus infection and genetic susceptibility in endemic areas of nasopharyngeal cancer, such as southern Asia and northeastern India. In line with the results of several previous studies (12, 46), the latest data demonstrate a negative correlation between the incidence of nasopharyngeal cancer and HDI.

Betel nut chewing has been identified as a significant risk factor for oropharyngeal cancer in Southeast Asia and among ethnic minority groups residing in this region (20, 47, 48). HPV has been identified as another major risk factor for oropharyngeal cancer. HNC exhibit a distinct socio-economic profile, with individuals from the poorest backgrounds experiencing the highest risk of developing the disease. However, this socio-economic risk cannot be fully explained by smoking and alcohol-abusing behaviors. A study has demonstrated that oral HPV infection is necessary for the sustained significant increase in the incidence of oropharyngeal cancer, the majority of which is observed in male patients. Prophylactic HPV vaccination holds considerable promise for reversing these incidence trends in the future (49).

The present study has identified several factors associated with an increased risk of salivary gland cancer, including a history of HNC and cervicofacial radiation therapy. In addition, tobacco use and alcohol consumption have been found to be unrelated to the risk of salivary gland cancer. Furthermore, industries such as grain and other crop production, furniture manufacturing, intercity highway transportation, and industrial cleaning have been identified as contributing to an elevated risk of salivary gland cancer (50).

Regional disparities are closely related to racial disparities. Recognizing how risk factors influence the age of initial cancer diagnosis may lead to more suitable screening and prevention strategies.

In most countries and regions, there is a persistent gender gap in the prevalence and mortality rates of HNC, reflecting the strong association between sexuality and exposure to risk factors. This discrepancy can be attributed, in large part, to the higher rates of smoking and alcohol consumption among males In industrialized countries, men are affected two to three times as often as women, largely due to higher use of alcohol and tobacco (51). Notably, female patients have exhibited higher survival rates, particularly in cases of oral cavity tumors. This finding has been replicated in numerous studies (52–54). One hypothesis posits that this disparity in survival may be attributed, at least in part, to the greater stability of social support networks available to women, who are more likely to receive an early diagnosis and access timely treatment. These observations underscore the potential value of enhancing social support for male patients to improve their survival outcomes.

According to the most recent statistics for 2022, the global burden of HNC remains significant. Among the six types of HNC, lip and oral cancer account for more than two-fifths of both incidence cases and deaths, underscoring the need for focused attention. This heightened attention may be attributable to the diversity of lip and oral cavity cancer and the increasing detection of lip and mouth cancers early on, largely driven by heightened awareness of oral hygiene and the development of the economy. It is estimated that two-thirds of the more than 400,000 new cases of oral cancer diagnosed each year occur in Asian countries such as Sri Lanka, Indonesia, India, Pakistan and Bangladesh (55).

Prognosis for oral cancer is generally considered poor, with patients playing a significant role in delays in diagnosis (56). The overall 5-year survival rate for oral cancer is as low as 40%, but if diagnosed at an early stage, the survival rate can exceed 80%. However, 50% of oral cancers are diagnosed at a late stage because most patients have no symptoms in the early stages and do not seek medical attention until they experience pain and bleeding (57). The present study underscores the necessity of implementing early screening measures for individuals across various age groups. A salient finding of our study is the observed increase in the incidence of lip and oral cavity cancer among individuals older than 25 years. Consequently, it is imperative to prioritize the development of screening programs specifically tailored to this demographic.

The global impact of cancer is unequally distributed, with persistent and widening disparities related to socioeconomic factors (58). Noncommunicable diseases, including alcohol use, tobacco, unhealthy diets, and physical inactivity, are the leading causes of death worldwide and are significant risk factors for the development of HNC, which disproportionately affects individuals in low-income and lower-middle-income countries (59). Conversely, in high-income countries, individuals with higher socioeconomic status are less susceptible to these conditions, suggesting a correlation between socioeconomic status and the prevalence of HNC in low-income and lower-middle-income countries, as evidenced by our study.

The prevalence of tobacco use has exhibited an upward trend in developing countries, concomitant with their economic advancement. Conversely, developed countries have witnessed a general decrease in the prevalence of tobacco use. However, there have been increases among specific populations, with smoking prevalence rising among certain groups, predominantly women. As discussed in our article, lip and oral cavity cancer is associated with social and economic status, with the highest incidence and mortality rates occurring in the most disadvantaged groups of the population, such as low HDI and moderate HDI countries. This association is particularly strong for men. However, a notable exception emerges among the younger population, with 25% of this group belonging to the professional class (57).

When interpreting these findings, particularly in comparisons across different HDI levels, it is crucial to acknowledge potential limitations in the quality of underlying data. Low-HDI countries often face challenges such as incomplete coverage of cancer registries, limited diagnostic capabilities, and absence of treatment and care (60, 61). These factors may lead to underreporting of incidence and mortality rates. To address data gaps in countries with low HDI scores and enhance reliability, the International Agency for Research on Cancer uses data from its cancer registry partners and collaborates with national staff to jointly improve data quality, coverage, and capabilities.

### 4.3 Future projections (to 2050)

Even a 3% annual decline in global prevalence would increase the projected burden of HNC. If prevalence remains unchanged from 2022, an additional 600,000 new cases would be expected in 2050.

Significant disparities are evident among various population subgroups, with socioeconomic and cultural factors playing a pivotal role in determining exposure to risk factors and variations in health outcomes. In addition, it is important to note that these imbalances are influenced by additional dimensions, including educational access, economic and access to healthy food, and adequacy of healthcare facilities (62). The phenomenon has been termed “healthy bullying” in the contemporary era. Research predicting the future burden of cancer suggests that, without of targeted policy measures to eradicate the sources of cancer, there will be an increase in inequality between socio-economic groups (62). The surge in cancer incidence and mortality is expected to be most significant in countries with low and medium HDI. The exacerbation of cancer in the coming decades is projected to be primarily driven by population growth, aging, and detrimental lifestyles. A significant portion of the global cancer burden is attributable to tobacco use, infection, and malnutrition. A recent study has demonstrated that the effective implementation of tobacco control policies at the domestic level is paramount in ensuring significant reductions in tobacco use going forward (35).

In order to counteract the projected rise in the absolute number of HNC cases and deaths, proactive and robust global public health responses are urgently needed. Firstly, it is vital to accelerate primary prevention by intensifying efforts to reduce key risk factors, including the enforced implementation of WHO Framework Convention on Tobacco Control measures. These include higher taxes, comprehensive advertising bans and plain packaging. Specific attention should be paid to curbing the rising smoking rates among women in low and middle HDI countries through targeted campaigns and stricter regulations on gendered marketing. Concurrently, there is a necessity to reinforce policies aimed at reducing harmful alcohol use, whilst simultaneously expanding global HPV vaccination programs. This expansion must be accompanied by a particular focus on overcoming access and cost barriers in low-HDI countries. This can be achieved via international support mechanisms, and through the integration of HPV vaccination programs into existing immunization platforms. Secondly, the expansion of early detection should be pursued through the implementation of widespread, cost-effective screening for orally visible cancers, particularly in high-incidence regions such as Asia. This can be achieved by training primary healthcare workers to perform visual inspections in community settings, facilitating diagnosis at earlier stages. Health systems in low and middle HDI countries should be strengthened through international investment to improve cancer registration, expand pathology services, and increase radiotherapy and chemotherapy availability and affordability. Finally, launching public awareness campaigns to educate on the signs of early HNC and risks associated with certain substances can encourage people to seek earlier care.

Overall, utilizing the GLOBOCAN data, this study provides a valuable opportunity to advance the global understanding of the epidemiology of HNC and to promote the advancement of more effective strategies for the prevention and control of HNC.

## 5 Limitation

Notwithstanding the dependability of the data sources and analysis, it must be acknowledged that our study has numerous limitations. Primarily, our study is limited by the aggregated nature of the GLOBOCAN database, which prevented a detailed subgroup analysis of vulnerable. This lack of granularity may obscure important disparities and limits the ability to tailor specific interventions. It is recommended that future studies take advantage of individual-level data or national registries with detailed demographic stratifications in order to explore these critical dimensions. Secondly, the quality of data from less developed countries may introduce bias due to unreliable data, potentially leading to inaccurate results. Particularly in the case of historical data, we had a single data source, the GLOBOCAN database, and incomplete data may have had a significant impact on the results; in the future, several databases can be combined for analysis. Additionally, while the HDI is a comprehensive measure of a country’s development, it doesn’t capture everything. Aspects of human development, such as education level and social equality. We also did not quantify the effect of age, sex, region, or combinations on HNCs, but studies may explore this in future. Furthermore, a considerable number of economically underdeveloped countries have not been adequately represented in the 30 years of accumulated data. This has significantly hindered our capacity to comprehensively assess and analyze historical data. Finally, we did not quantify the effect of age, sex, and geographic region or their combination on HNC incidence and mortality, and future studies could be conducted in this area.

## Data Availability

Publicly available datasets were analyzed in this study. This data can be found here: https://gco.iarc.fr/.
